# Improving Rice Modeling Success Rate with Ternary Non-structural Fertilizer Response Model

**DOI:** 10.1038/s41598-018-27323-2

**Published:** 2018-06-13

**Authors:** Juan Li, Mingqing Zhang, Fang Chen, Baoquan Yao

**Affiliations:** 1 Soil and Fertilizer Institute, Fujian Academy of Agricultural Science, Fuzhou, 350013 China; 2 Key Laboratory of Aquatic Botany and Watershed Ecology, Wuhan Botanical Garden, Chinese Academy of Sciences, Wuhan, 430074 China; 3 Wuhan Office of International Plant Nutrition Institute, Wuhan, 430074 China; 4 Fujian Cropland Construction and Soil and Fertilizer Station, Fuzhou, 350003 China

## Abstract

Fertilizer response modelling is an important technical approach to realize metrological fertilization on rice. With the goal of solving the problems of a low success rate of a ternary quadratic polynomial model (TPFM) and to expand the model’s applicability, this paper established a ternary non-structural fertilizer response model (TNFM) based on the experimental results from N, P and K fertilized rice fields. Our research results showed that the TNFM significantly improved the modelling success rate by addressing problems arising from setting the bias and multicollinearity in a TPFM. The results from 88 rice field trials in China indicated that the proportion of typical TNFMs that satisfy the general fertilizer response law of plant nutrition was 40.9%, while the analogous proportion of TPFMs was only 26.1%. The recommended fertilization showed a significant positive linear correlation between the two models, and the parameters *N*_0_, *P*_0_ and *K*_0_ that estimated the value of soil supplying nutrient equivalents can be used as better indicators of yield potential in plots where no N or P or K fertilizer was applied. The theoretical analysis showed that the new model has a higher fitting accuracy and a wider application range.

## Introduction

Paddy rice is one of the most important grain crops in China, and fertilizer plays a key role in rice production. However, over-fertilization is common in most rice-producing regions in China, which results in a low fertilizer use efficiency and non-point source nitrogen and phosphorus pollution^1^. Therefore, the study and popularization of metrological fertilization technology is a key approach to improve the fertilizer use efficiency to realize a high yield and high-quality rice production. The fertilizer response function method is currently the main technical method for metrological fertilization^2–7^. This method is based on field experiments with fertilizer to establish a statistical regression model between the fertilization rate and crop yield based on biostatistical principles, after which the recommended fertilization rate for the representative fields can be calculated. This fertilization model is used to directly “inquire” the crops, and its measurement accuracy and validity are better than that of other methods^8^.

Because paddy rice production is characterized by highly decentralized management in China, the fertilizer response function to metrological fertilization is advantageous because it is intuitive, conveniently popularized and applied in rural villages, which is the main technical approach used to realize the rational fertilization of rice^8,9^. A quadratic polynomial model has been mostly studied and applied in many fertilizer response models^6,7,10–12^. However, many studies have shown that the typical proportion of a unary quadratic polynomial fertilizer response model that meets with the general fertilizer response law of plant nutrition was only about 60%, while the probability for a binary quadratic polynomial fertilizer response model was only 40.2%^13,14^, and the probability for a ternary quadratic polynomial fertilizer response model (TPFM) was as low as 23.6%^15^.

Quadratic polynomial fertilizer response models generate many non-typical models during model establishment, which severely reduces the accuracy of computation and practical value. Researchers worldwide have deeply studied and proposed many ideas for improvement^7^, but related issues still exist. Such studies and improvement measures rarely reported the specification bias in the fertilizer response model itself and the suggestions for improvement. Studies show that there are many problems such as specification bias and multicollinearity in unary, binary and tertiary quadratic polynomial fertilizer response models and other similar polynomial models^7^, which have led to a low modelling success rate. Zhang *et al*.^16^ established a unary non-structured fertilizer response model that well overcame the model specification defect. Compared with a quadratic polynomial fertilizer response model, the new model improved the fitting precision, expanded its applicability and reduced the recommended fertilization rate.

There is a significant increase in yield applying N, P, K fertilizers respectively in paddy rice production in China. Because of their interaction effect in N, P, K fertilizer, ternary fertilizer response model can more accurately calculate the recommended application rate of fertilizer. Therefore, the authors discuss the construction method of a tertiary non-structural fertilizer response model (TNFM) based on a unary non-structural fertilizer response model and the effect on the fitting of recent N, P and K fertilizer experimental data in rice fields in this paper. The objective is to expand the applicability of ternary fertilizer response models and to improve the modelling success rate to provide a new method for the N, P and K metrological fertilization of paddy rice.

## Results

### The effect of TPFM on the fitting of the experimental data

The mathematical expression of a TPFM is below:

1 $${\rm{Y}}={b}_{0}+{b}_{1}{\rm{N}}+{b}_{2}{\rm{P}}+{b}_{3}{\rm{K}}+{b}_{4}{{\rm{N}}}^{2}+{b}_{5}{{\rm{P}}}^{2}+{b}_{6}{{\rm{K}}}^{2}+{b}_{7}{\rm{NP}}+{b}_{8}{\rm{NK}}+{b}_{9}{\rm{PK}}$$

where Y is the fitting crop yield; N, P, and K are the application rates of N, P_2_O_5_, or K_2_O fertilizer; and *b*_0_ to *b*_9_ are the fertilizer response coefficients. According to the application rate of N, P, K fertilizer and yield for the treatments in Table 1, we may build a TPFM by using the ordinary least square (OLS) method, as shown in Table 2. The results showed that the fertilizer response model based on the high soil fertility in Xianyou County failed the significance test and loses its application value, while the other models of 5 sites were statistically significant.

**Table 1:** Early rice production in field experiments at different soil fertility levels.

Treatments	Grain yield in Pinghe County (kg/hm^2^)			Grain yield in Xianyou County (kg/hm^2^)		
	1. Low fertility	2. Medium fertility	3. High fertility	4. Low fertility	5. Medium fertility	6. High fertility
(1) N_0_P_0_K_0_	4320 ± 91	5180 ± 518	5835 ± 325	4858 ± 162	5415 ± 288	6375 ± 457
(2) N_0_P_2_K_2_	5325 ± 153	6375 ± 778	6835 ± 458	5520 ± 436	5907 ± 382	7080 ± 838
(3) N_1_P_2_K_2_	6120 ± 494	6995 ± 576	7675 ± 621	6568 ± 553	6741 ± 633	7587 ± 562
(4) N_2_P_0_K_2_	5985 ± 744	7115 ± 348	7905 ± 1105	6654 ± 555	6831 ± 608	7533 ± 536
(5) N_2_P_1_K_2_	6390 ± 716	7155 ± 131	8090 ± 1089	7031 ± 665	7302 ± 678	7767 ± 702
(6) N_2_P_2_K_2_	6581 ± 744	7190 ± 90	8465 ± 1218	7331 ± 483	7689 ± 574	8025 ± 697
(7) N_2_P_3_K_2_	6559 ± 563	7330 ± 128	8480 ± 1330	7078 ± 494	7236 ± 760	7926 ± 748
(8) N_2_P_2_K_0_	5760 ± 551	7010 ± 310	8040 ± 1001	6416 ± 601	6495 ± 791	7965 ± 483
(9) N_2_P_2_K_1_	6176 ± 671	7120 ± 111	8180 ± 998	6917 ± 424	7326 ± 665	7563 ± 538
(10) N_2_P_2_K_3_	6270 ± 814	7270 ± 230	8525 ± 1201	7005 ± 443	7107 ± 605	7770 ± 529
(11) N_3_P_2_K_2_	6326 ± 611	7395 ± 541	8460 ± 1182	6754 ± 640	6774 ± 646	7794 ± 468
(12) N_1_P_1_K_2_	6165 ± 690	7295 ± 150	8280 ± 1081	6538 ± 550	6717 ± 766	7617 ± 613
(13) N_1_P_2_K_1_	6113 ± 603	7185 ± 303	8025 ± 754	6675 ± 547	6957 ± 925	7962 ± 631
(14) N_2_P_1_K_1_	6304 ± 732	7215 ± 278	8125 ± 979	6763 ± 573	6930 ± 510	7827 ± 302

Note: The subscript “2” in the treatment designations indicates the local N, P_2_O_5_ and K_2_O recommended fertilization rate. The application rates of N-P_2_O_5_-K_2_O were 165-75-105 kg/hm^2^ in Pinghe County, and 165-56-109 kg/hm^2^ for low or medium soil fertility and 165-53-112 kg/hm^2^ for high soil fertility in Xiuyou County. The subscript “0” in the treatment designations indicates no fertilization, and the subscripts “1” and “3” in the treatment designations indicate 50% and 150% of the “2” level. The yield data in the table are expressed as the average value ± standard deviation of 3 repetitions in Pinghe County and 4 repetitions in Xianyou County.

**Table 2:** Regression modelling of TPFMs by the OLS method and its typicality discriminant.

Sites No.	Parameters of the model (1)										Statistical test			Typicality discriminant		
	*b*_0_	*b*_1_	*b*_2_	*b*_3_	*b*_4_	*b*_5_	*b*_6_	*b*_7_	*b*_8_	*b*_9_	*F*	*R* ^2^	*P*	PS	Max	RF
1	4337	12.964	8.203	14.483	−0.0516	−0.0719	−0.0814	0.0139	0.0027	0.0190	30.4**	0.986	0.002	Y	Y	Y
2	5225	8.335	14.839	16.526	−0.0266	−0.0531	−0.0404	0.0269	−0.0019	−0.1150	8.2*	0.949	0.029	Y	N	—
3	5886	10.491	12.832	17.672	−0.0441	−0.0838	−0.0302	0.0935	−0.0015	−0.1364	10.1*	0.958	0.020	Y	N	—
4	4855	15.311	18.921	6.795	−0.0576	−0.1339	−0.0553	−0.0052	0.0423	−0.0172	45.9**	0.990	0.001	Y	Y	Y
5	5392	13.732	19.479	5.950	−0.0605	−0.1553	−0.0760	−0.0113	0.0592	0.0072	7.1*	0.941	0.038	Y	Y	Y
6	6404	8.508	28.168	1.112	−0.0277	−0.1043	−0.0110	−0.0413	0.0285	−0.0833	4.6	0.911	0.079	—	—	—

Note: “PS” indicates unreasonable parameter symbols, “Max” indicates no maximum yield point, “RF” indicates extrapolative recommended fertilization rate. “Y” indicates normal, and “N” indicates abnormal. “—” means no correlated calculation, because of belonging to no-typical model such as Site 2 and Site 3 or failure to pass the significance test such as Site 6.

The model’s typicality discrimination^15^ showed that the TPFMs were based on trial site 1 with low soil fertility in Pinghe county, trial site 4 with low soil fertility and trial site 5 with medium soil fertility in Xianyou county, and show a typical fertilizer response model, which can be used to recommend fertilization by the marginal product derivative method. However, for the TPFMs based on trial sites with medium or high soil fertility in Pinghe county, although the algebraic sign of the model parameter was reasonable, there is not global maximum output point occurred in the model. These showed non-typical fertilizer response models, which could not be used to recommend a fertilization scheme. The results showed that TPFM has a lower fitting ability for the results of the rice field experimental response to N, P, and K fertilization.

### The fitting effect of TNFM on the experimental data

To address the problems of specification bias and multicollinearity in a TPFM^8^, Zhang *et al*.^16^ established a unary non-structural fertilizer response model based on the results of single factor field experiments involving N, P and K fertilization of rice:

2 $$Y=A({s}_{0}+{\rm{X}}){e}^{-cX}$$

where Y is the crop yield; X is the application rate of N, P or K fertilizer; *s*_0_ is the equivalent of nutrients supplied from the soil; *c* is the yield coefficient of fertilization; and *A* is the conversion coefficient of soil fertility to rice yield at X = 0.

In model (2), the crop yield must be zero when both the fertilizer application rate and the soil nutrient supply equivalent are equal to zero. Therefore, according to the principle of the irreplaceable function of plant nutrient elements, a ternary non-structural fertilizer response model (TNFM) can be described by:

3 $$Y=A({N}_{0}+N)({P}_{0}+P)({K}_{0}+K){e}^{-{c}_{1}N-{c}_{2}P-{c}_{3}K}.$$

where *N*_0_, *P*_0_, and *K*_0_ are the soil nutrient supply equivalents of N, P_2_O_5_ and K_2_O, respectively, and *c*_1_, *c*_2_ and *c*_3_ are the increase yield effect coefficient of nitrogen, phosphorus and potash fertilizer. The parameter *A* is the conversion coefficient of soil fertility to rice yield when the application rates of nitrogen, phosphorus and potassium fertilizer are equal to zero, and the meaning of other algebraic symbols is the same as in model (2).

In order to study the application effect of the TNFM, we used the experimental results in Table 1 in a regression by model (3) in Table 3. Statistical testing indicated that all of the TNFMs based on the 6 trial sites were statistically significant. Moreover, the model’s statistical significance probability values (*P*) were significantly smaller than the corresponding indices in Table 2. In particular, the *P* value of site 6 was reduced to 0.000 and was significant, while the *P* value of model (1) was 0.079 and not significant.

**Table 3:** Regression analysis and typicality discriminant of TNFM.

Sites No.	Parameters of model (3)							Statistical test			Typicality discriminant		
	${\boldsymbol{A}}{\boldsymbol{\times }}1{{\bf{0}}}^{{\bf{3}}}$	*N*_0_	*P*_0_	*K*_0_	${\boldsymbol{c}}\,{}_{1}\,{\boldsymbol{\times }}\,\,1{{\bf{0}}}^{{\bf{3}}}$	${\boldsymbol{c}}\,{}_{2}\,{\boldsymbol{\times }}\,\,1{{\bf{0}}}^{{\bf{3}}}$	${\boldsymbol{c}}\,{}_{3}\,{\boldsymbol{\times }}\,\,1{{\bf{0}}}^{{\bf{3}}}$	*F*	*R* ^2^	*P*	PS	Max	RF
1	1.1614	163.33	171.25	133.45	3.0033	3.8371	4.3251	67.6**	0.983	0.000	Y	Y	Y
2	1.0277	187.69	152.99	182.79	2.7800	4.4679	3.5317	13.2**	0.919	0.002	Y	Y	Y
3	6.8048	166.15	162.17	323.52	2.8394	4.0805	2.0920	19.6**	0.944	0.000	Y	Y	Y
4	0.9691	137.97	177.34	202.35	3.2818	3.9354	3.1508	47.4**	0.976	0.000	Y	Y	Y
5	0.8582	156.21	226.08	173.26	3.1919	3.1841	3.7170	9.4**	0.889	0.005	Y	Y	Y
6	0.5323	221.48	135.06	407.43	2.6163	5.0518	2.0577	9.4**	0.890	0.005	Y	Y	Y

Note: “PS” means unreasonable parameters symbols, “Max” means no maximum yield point, “RF” means extrapolative recommended fertilization rate. “Y” means normal, “N” means abnormal.

The results for the model typicality discriminant^15^ in Table 3 show that the data for site 6 by model (1) were nonsignificant and the data for sites 2 and 3 were assigned to a non-typical model and were converted into a typical model by the TNFM. The models of sites 1, 4 and 5 were typical by model (1), and the modelling results by model (3) are also typical models.

### Recommended fertilization rates of TNFM

According to the analysis of mathematical theory, there is a peak rice yield of model (3) at a particular fertilization rate, corresponding to the fertilization rate that gave the maximum yield. Therefore, according to the principle of calculus, we can order the derivative of rice yield Y with respect to N, P and K in model (3) to be zero and can obtain the formula for the fertilization rate for the maximum yield:

4 $$\{\begin{array}{c}{N}_{max}=\frac{1}{{c}_{1}}-{N}_{0}\\ {P}_{max}=\frac{1}{{c}_{2}}-{P}_{0}\\ {K}_{max}=\frac{1}{{c}_{3}}-{K}_{0}\end{array}$$

We can command the derivative of rice yield Y with respect to N, P and K in model (3) to be the price reciprocal proportion of rice and fertilizer and obtain the calculation formula for the fertilization rate for the economic yield.

5 $$\{\begin{array}{c}{N}_{{\rm{e}}{\rm{c}}{\rm{o}}}=\frac{1}{{c}_{1}+\alpha /{Y}_{{\rm{e}}{\rm{c}}{\rm{o}}}}-{N}_{0}\\ {P}_{{\rm{e}}{\rm{c}}{\rm{o}}}=\frac{1}{{c}_{2}+\beta /{Y}_{{\rm{e}}{\rm{c}}{\rm{o}}}}-{P}_{0}\\ {K}_{{\rm{e}}{\rm{c}}{\rm{o}}}=\frac{1}{{c}_{3}+\gamma /{Y}_{{\rm{e}}{\rm{c}}{\rm{o}}}}-{K}_{0}\end{array}$$

where *a* = P_N_/P_Y_, *β* = P_P_/P_Y_, *γ* = P_K_/P_Y_, P_N_, P_P_, P_K_ and P_Y_ are the market price of N, P_2_O_5_ and K_2_O nutrients and grain per kg, respectively. *Y*_eco_ is the economic output. Experience shows that the difference in the maximum yield *Y*_max_ and the economic yield *Y*_eco_ from the fertilizer response model is very small, and *Y*_eco_ can be replaced by *Y*_max_ that is calculated from model (3). A refined calculation result of model (5) could also be obtained by the use of an iterative algorithm approach for calculation. Generally, 3~5 iterations are enough.

The maximum fertilization rates and the economical fertilization rates of N, P and K were calculated in Table 4 according to the estimated values of the parameters in the TNFM in Table 3 and models 4 and 5. The results show that the recommended fertilization rates for trial sites 2, 3 and 6, were all in the range of the fertilization rate of the experimental design, and no abnormal rate was noticed. The recommended fertilization rates have been calculated in Table 4 for trial sites 1, 4 and 5 and are typically modelled by model (1) or model (3). The results show little difference between the maximum fertilization rates or the economic fertilization rates for the two models, which indicates that the recommended fertilization rates should be reliable.

**Table 4:** Recommended application rates of the TNFM and TPFM.

Sites No.	TNFM models (kg/hm^2^)								TPFM models (kg/hm^2^)							
	Max. application rate				Economic application rate				Max. application rate				Economic application rate			
	N	P_2_O_5_	K_2_O	Yield	N	P_2_O_5_	K_2_O	Yield	N	P_2_O_5_	K_2_O	Yield	N	P_2_O_5_	K_2_O	Yield
1	170	89	98	6510	136	65	79	6423	174	87	102	6562	144	65	84	6478
2	172	70	100	7425	138	55	75	7339	—	—	—	—	—	—	—	—
3	186	83	154	8544	157	66	95	8421	—	—	—	—	—	—	—	—
4	166	77	115	7090	141	56	83	6997	174	60	119	7154	143	53	85	7071
5	157	88	96	7283	130	57	73	7189	159	59	104	7368	128	52	75	7289
6	161	63	79	7958	124	51	133	7827	—	—	—	—	—	—	—	—

Note: “—” means no correlated calculation, because TPFM is belonging to no-typical model such as Site 2 and Site 3 or failure to pass the significance test such as Site 6.

### Fitting effect evaluation of the TNFM

The results of small samples in Tables 2 and 3 show that model (3) has a higher fitting accuracy and a wide application scope. In order to more accurately evaluate the reliability and application value of the TNFM, the authors collected 88 rice field experimental results with a “3414” design conducted in the Guangxi, Guangdong, Fujian, Jiangxi, Hunan, Hubei, Anhui, Jiangsu and Zhejiang provinces of China over the past 10 years. We set up a one by one fertilizer response model for each experimental site using model (1) and model (3). The statistical results in Table 5 show that the proportion of a typical model for TPFM is only 26.1%. However, with TNFM, the proportion of a typical model increased to 40.9%, improving by 14.8 percentage points. Therefore, the new model had a significantly improved modelling success rate.

**Table 5:** Fitting effect of the TNFM compared with the TPFM for the fertilization response to N, P and K in rice

Models	Experimental No.	NRSS (%)	Ratio of statistical significance in the models (%)			
			Non-typical models			Typical models
			PS	Max	RF	
TPFM	88	18.2 (16)	23.9 (21)	14.8 (13)	17.0(15)	26.1 (23)
TNFM	88	10.2 (9)	2.3 (2)	0.0 (0)	46.6 (41)	40.9 (36)

Note: “NRSS” indicates a nonsignificant fertilizer response model. The number in the bracket means the number of trials. “PS” means unreasonable parameters symbols, “Max” means no maximum yield point, “RF” means extrapolative recommended fertilization rate.

A further analysis also showed that the TNFM significantly reduces the proportion of nonsignificant models or those that have an unreasonable coefficient algebraic sign. Meanwhile, the proportion of non-typical model types that did not have a maximum yield point was zero. However, the TNFMs significantly increased the proportion of the non-typical models that were extrapolated to recommend application rate compared with TPFMs. This result showed that there was a rational difference among the non-typical model types that did not have a maximum yield point and the extrapolated application rate between the two models.

**Figure 1:**
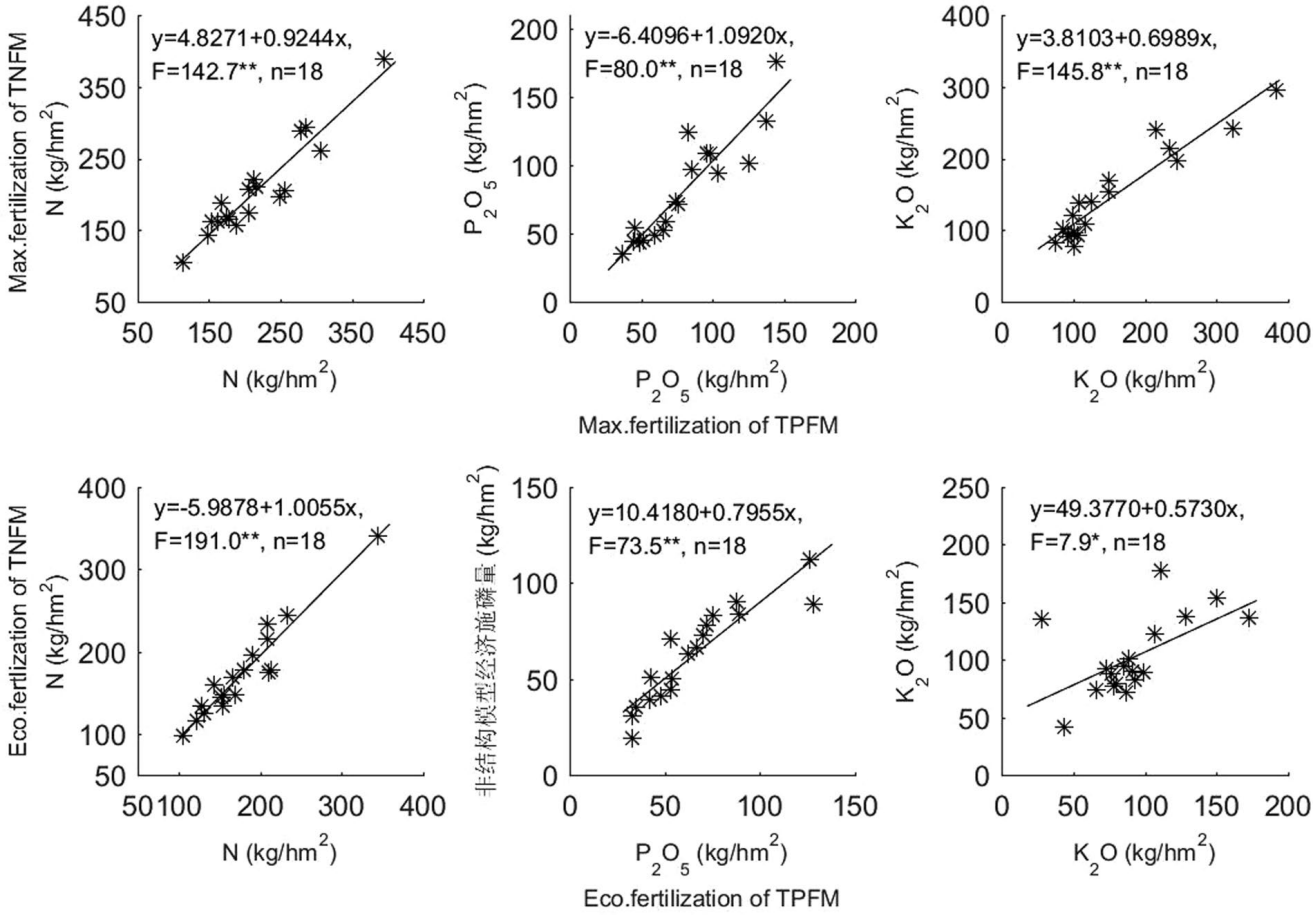
Correlation analysis of the recommended fertilization rate by the TNFM and TPFM.

A typical model was obtained for 18 experimental sites using the two models in 88 field experiments. The correlation analysis in Fig. 1 shows that a highly significant positive linear correlation was present between the two models for both the maximum fertilization rate and the economic fertilization rate for N, P and K, which indicates that the new model has good inheritance and reliability with the recommended fertilization rate.

**Figure 2:**
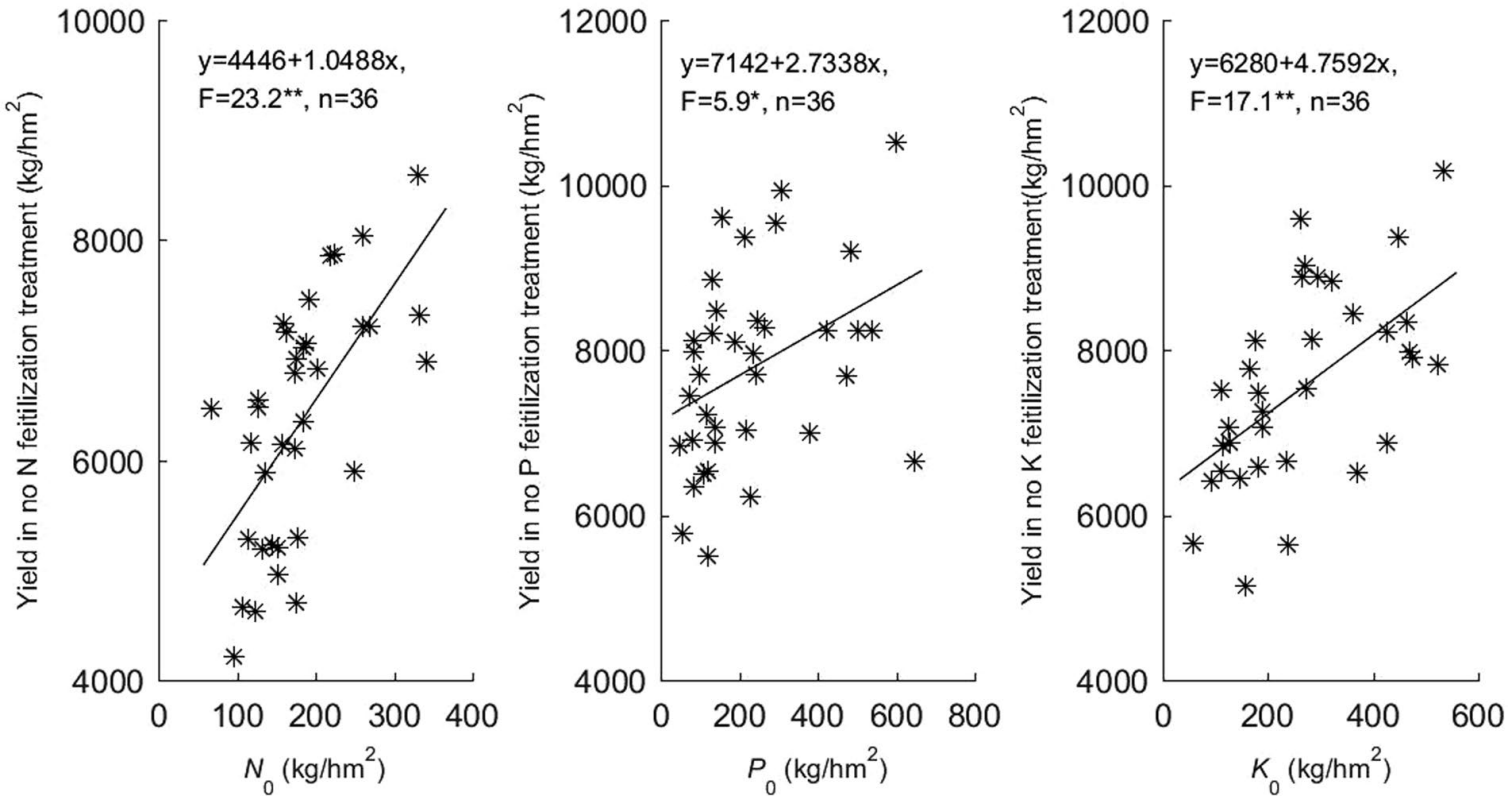
Correlation analysis between *N*_0_, *P*_0_, *K*_0_ and rice output for the no N or no P or no K fertilization treatments.

More interesting is that the soil nutrient supply equivalent *N*_0_, *P*_0_ and *K*_0_ that was estimated by model (3) has a significant positive linear correlation with rice output for the treatments with no N fertilization, no P fertilization and no K fertilization (Fig. 2), which showed that the estimated value of the soil nutrient supply equivalent of *N*_0_, *P*_0_ and *K*_0_ by the new model better reflected the paddy soil supply potential of N, P and K.

## Discussion

### Model specification bias of TPFM and its consequences

The response to N, P, and K fertilization in China’s rice planting areas in 88 rice field experiments shown in Table 5 indicated that a typical model occurred for the TPFM at only 26.1%. The excessive low modelling success rate casts doubt on the rationality of the model setting itself.

A theoretical analysis shows that a unary quadratic polynomial fertilizer response model and a binary or ternary quadratic polynomial model developed from the unary model assume a linear relationship between the increased crop yield rate per unit of nutrition and fertilizer application, which leads to a fertilizer efficiency that has a symmetric relationship^7^ both before and after the maximum application rate. This model setting ignored crop fertilizer response characteristics that of new high-yielding variety that have been popularized and applied extensively and display tolerance to over-fertilization, so leading great alleviation of yield reduction than with other varieties. It also ignored the effect of the soil nutrient buffer capacity and the negative effect of over-fertilization on crop yield. Therefore, the model setup of the quadratic polynomial fertilizer response model used commonly at present does not conform to the theoretical assumption that the regression model is unbiased in a classical linear regression analysis^17^. Meanwhile, the regression variables of the quadratic polynomial fertilizer response model are strongly multicollinear^7^, which seriously restricts the validity of regression modelling by OLS and the reliability of statistical tests. Therefore, the model setting bias and multicollinearity are important reasons that might have led to the low success rate of the ternary quadratic polynomial models.

Statisticians have proposed many biased estimation methods to deal with the multicollinearity problem in polynomial statistical models, such as ridge regression, principal component regression, and partial least-squares regression^17,18^, to eliminate or reduce the dangers of multicollinearity. However, biased estimation fails to solve the setting bias problem for the fertilizer response model itself.

### The applicability of the TNFM

Many mechanistic models for the soil-crop root nutrient absorption process^19,20^ or semi-mechanistic and semi-empirical models^12,21–23^ have been proposed as crop metrological fertilization models to account for the effects of agricultural fertilization and the soil nutrient supplying capacity. These research results have important scientific value to aid in the understanding and mastery of the crop nutrient absorption process and in the identification of factors that influence and control technology, etc. However, these two types of models require many parameters, some of which are difficult to measure, and the practicability of the two models is deficient for a highly decentralized agricultural production pattern. While based on crop fertilization rate and yield effects, unary and multivariate statistical models have the advantages of simplicity and practicality and have been widely studied and popularized^4,5,10,24^. But, it is unfortunate that this polynomial model has problems such as bias error and multicollinearity^7^, which leads to a significantly lower modelling success rate.

We propose a ternary non-structural fertilizer response model that assumes a non-liner relationship for the increase in crop yield per unit of nutrition and fertilizer application to overcome the fixed error of a polynomial fertilizer response model. The new model cannot be directly linearly transformed, which better overcomes the problem of multicollinearity. In the 88 field experiments, the proportion of typical models obtained by the TNFM was 40.9%, which is 1.6-fold greater than with the TPFM. The new model has a higher fitting accuracy and a wider application scope (Table 3). Correlation analysis shows that the maximum fertilization rate or economic fertilization rate recommended by the new model has a significant positive linear correlation with those estimated by the TPFM (Fig. 1).

The new model’s estimates for *N*_0_, *P*_0_ and *K*_0_ have a significant positive linear correlation with the corresponding grain yield in a nutrient-deficient area (Fig. 2), which indicates that the estimated value of soil nutrient-supply equivalent better reflects the potential of the paddy soil nutrient-supply of nitrogen, phosphorus and potassium and provides a new technical method and index for evaluating paddy soil nutrient-supplying ability and guiding the rational fertilization of paddy rice. The statistical results in Table 5 showed that the recommended fertilization rate by the new model that the proportion of the non-typical model belong to extrapolating the recommended fertilization was higher than that of the quadratic polynomial fertilizer response model. It indicated that the TNFM has a higher request of the fertilization rate design in order to reduce the ratio of the extrapolation model. Fortunately, this requirement is easy to do in experimental design.

A Taylor expansion gives ${{e}}^{{x}}=1+{x}+G({x}),{x}\in (\,-\,\infty ,\,+\,\infty )$, where ${\rm{G}}({x})=\frac{{{x}}^{2}}{2!}+\cdots +\frac{{{x}}^{n}}{n!}+\cdots .$ In the TNFM model, the parameters *c*_1_, *c*_2_ and *c*_3_ are at the 10^−3^ order level (Table 3); if only the first two items of expansion are considered, model (3) becomes: Y = *A*(*N*_0_ + *B*N − *c*_1_N^2^)(*P*_0_ + *C*P − *c*_2_P^2^)(*K*_0_ + *D*K − *c*_3_K^2^), where *B* = 1 − *N*_0_*c*_1_, *C* = 1 − *P*_0_*c*_2_ and *D* = 1 − *K*_0_*c*_3_. Expanding the algebraic expression, and ignoring the product items in pairs among *c*_1_, *c*_2_ and *c*_3_, the product terms of *c*_1_*c*_2_*c*_3,_ and the three factor interactions of N, P and K allows model (3) to be transformed to: Y = *A* (*N*_0_*P*_0_*K*_0_ + *BP*_0_*K*_0_N + *CN*_0_*K*_0_P + *DN*_0_*P*_0_K − *c*_1_*P*_0_*K*_0_N^2^ − *c*_2_*N*_0_*K*_0_P^2^ − *c*_3_*N*_0_*P*_0_K^2^ + *BCK*_0_NP + *BDP*_0_NK + *CDN*_0_PK). This result has the same mathematical form as model (1). It can be seen that, when the effect of the above ignored items is small enough in some experimental results, both model (1) and model (3) show a good fitting effect. On the contrary, the ternary quadratic polynomial model cannot fit well due to oversimplification, but the TNFM better fits the relevant trial results due to no such simplification. Therefore, the TPFM is a simplified and special case of the TNFM, and the new model has wider application scope.

## Conclusion

A ternary non-structural fertilizer response model can overcome the model specification bias and multicollinearity of a quadratic polynomial model, which significantly improved the model’s fitting accuracy and success rate in rice field experiments. A theoretical analysis showed that the TPFM is a simplified and special case of the TNFM, and the new model has higher fitting accuracy and wider application scope.

## Materials and Methods

### N, P and K fertilizer experimental design for rice field experiments

Field experiments to measure the early rice response to N, P and K were carried out in the main paddy rice production regions of Xianyou County and Pinghe County in Fujian province during 2015 and 2016. The experiment used a “3414” design^25^: (1) N_0_P_0_K_0_, (2) N_0_P_2_K_2_, (3) N_1_P_2_K_2_, (4) N_2_P_0_K_2_, (5) N_2_P_1_K_2_, (6) N_2_P_2_K_2_, (7) N_2_P_3_K_2_, (8) N_2_P_2_K_0_, (9) N_2_P_2_K_1_, (10) N_2_P_2_K_3_, (11) N_3_P_2_K_2_, (12) N_1_P_1_K_2_, (13) N_1_P_2_K_1_, (14) N_2_P_1_K_1_. The subscript “2” indicates the local N, P or K recommended fertilization rate. The subscript “0” indicates no fertilization, and the subscripts “1” and “3” indicate 50% and 150% of the “2” level, respectively. The field experiment plot size was 20 m^2^ with three replications and a randomly arranged block. Local main rice varieties were selected as the experimental varieties. Urea (N 46%), calcium superphosphate (P_2_O_5_ 12%), and potassium chloride (K_2_O 60%) were used as experimental fertilizers. The fertilizers for basal dressing included all of the P_2_O_5_, 50% of the N and 50% of the K_2_O, and approximately 40% of the N was applied as a top-dressing at the tillering stage and another 10% of the N and 50% of the K_2_O was applied as a top-dressing at the heading stage. At harvest, the fresh weight and dry weight of the rice straw and the grain in each plot were measured separately. Other field management activities were carried out according to common practice for the location.

Soil samples were taken before the field experiments. The soil samples were tested by conventional methods^26^. The soil pH was measured with a potentiometer, the soil organic matter was measured by a volumetric method with potassium dichromate, the available N was measured using an alkaline hydrolysis diffusion method, the available P was measured using 0.5 mol/L sodium bicarbonate with a lixiviation-Mo-Sb anti-spectrophotometer, and the available K was measured using 1 mol/L ammonium acetate with a lixiviation-flame photometer. The main physical and chemical properties of the observed soils are shown in Table 6.

**Table 6:** Main physical and chemical properties of experimental soils in early rice.

No	Experimental sites	Soil fertility	Soil physical and chemical properties				
			pH	OM (g/kg)	Alkali-hydr. N (mg/kg)	Olsen-P (mg/kg)	Avail. K (mg/kg)
1	Pinghe county	low	4.90	27.24	121.5	28.4	67.0
2	Pinghe county	medium	4.87	29.75	156.0	29.1	61.3
3	Pinghe county	high	4.90	32.74	188.7	38.5	85.0
4	Xianyou county	low	5.40	18.62	112.0	16.6	41.9
5	Xianyou county	medium	5.24	25.94	151.5	20.8	57.2
6	Xianyou county	high	5.46	24.54	148.2	24.7	65.0

### Rice field data collection for N, P and K fertilization experiments with a “3414” design in China

In order to better evaluate the fitting ability of the TNFM response to N, P and K fertilization in rice, we collected published data from rice N, P and K fertilization field experiments that had a “3414” design in China in the past 10 years. We used the phrases “3414” and “rice” as the keywords of the thesis or abstract to search in the Tsinghua Tongfang (THTF) database. A total of 79 scientific papers were found, including 88 experiments that had soil sample test data, 14 fertilizer application rate treatments and associated yields with three replications. The source of the experimental data cited in this paper is shown in Table 7.

**Table 7:** Data from field experiments of the rice response to N, P and K fertilization with a “3414” design in China.

Provinces	Number of trials	Data source journal
Guangxi	14	Journal of Guangxi Agriculture, 2008, 23(6): 9–13; 2008, 23(6): 9–13; 2009, 24(4): 13–17; 2011, 26(2): 10–13; 2011, 26(4): 4–7; 2012, 27(3): 11–13. Guangxi Agricultural Sciences, 2007, 38(5): 541–543; Modern Agricultural Science and Technology, 2009, 7: 144–146; South China Agriculture, 2010, 1: 55–57; Jilin agriculture, 2011, 7: 88–90; Agricultural science and technology newsletter, 2011, 9: 48–51; Acta Agriculture Jiangxi, 2012, 24(11): 85–87; Agriculture & Technology, 2016, 36(23): 131–132.
Fujian*	17	Fujian rice and wheat technology, 2010, 28(2): 17–19; 2012, 30(40): 27–30; 2012, 30(1): 23–27; 2014, 32(1): 21–22; 2014, 32(3): 25–27. Fujian Agricultural Science and Technology,2011, 3:69–71; 2011, 2: 67–68; 2012, 2: 39–41; 2013, 1–2: 88–90; 2013, 3: 54–55. Acta Agriculture Jiangxi, 2009, 21(8): 68–69; 2009, 21(4): 30–32. Anhui Agricultural Science Bulletin, 2009, 15(16): 76–78; 2010, 16(20): 58–60. Shanghai Agricultural Science and Technology, 2007, 3, 35–36; Modern Agricultural Science and Technology, 2009, 19: 28–29; Tillage and Cultivation, 2012, 1: 31–32.
Zhejiang	12	Journal of Zhejiang Agricultural Sciences, 2010, 4: 799–800; 2010, 4: 784–786; 2010, 5: 982–984; 2010, 5: 982–984; 2010, 5: 982–984; 2011, 3: 563–565 Modern Agricultural Science and Technology, 2008, 4: 123–124; 2011, 21: 72–73; 2014, 24: 15–16; Jilin agriculture, 2010, 6: 98; Inner Mongolia agricultural science and technology, 2011, 3: 51–52; Shanghai Agricultural Science and Technology, 2016, 2: 85–86
Anhui	19	Anhui Agricultural Science Bulletin, 2007, 13(5): 116–118; 2007, 13(13): 146–147; 2008, 14(13): 79–81; 2009, 15(14): 87–91; 2009, 15(21): 97–98; 2010, 16(9): 75–79; 2011, 17(15): 81–82; 2012, 18(16): 82–84; 2012, 18(07): 108–109; 2014, 20(1–2): 42–44 Modern Agricultural Science and Technology, 2009, 13: 11–12; 2008, 14:143–144; 2013, 4:25–27; 2016, 4:9–11; 2015, 2:32–35; 2010, 31(9): 1 90–192; 2016, 22(05): 33–35 Gardening and seedling 2012, 5: 1–3, 17; Anhui Agricultural Sciences, 2014, 42(36): 12873–12874
Hunan	6	Hunan Agricultural Sciences, 2009, 3: 58~59, 61; 2015, 8: 60–62; 2015, (9): 33–34, 37 Modern Agricultural Sciences, 2009, 16(5): 98–99; 2011, 18(13): 51–52 Agricultural science and technology newsletter, 2010, 10: 46–48
Hubei	11	Agricultural science and technology newsletter, 2008, 47(12): 1416–1419; 2010, 49, 2008 (supplementary issue): 86–88, 89–92; 2011, 50(24): 5067–5071
Guangdong	2	Guangdong Agricultural Sciences, 2009, 4: 54–56; 2011, 15: 43–45
Jiangxi	2	Hebei agricultural technology, 2008, 2: 50; Modern Agricultural Technology, 2011, 3: 55
Jiangsu	1	Barley and Cereal Sciences, 2010, 2: 36–39
Guizhou	1	Guizhou Agricultural Sciences, 2008, 36(4): 43–45
Henan	1	China Agricultural Technology Extension, 2010, 26(3): 37–38
Liaoning	2	Beijing Agriculture, 2009, 3: 38–40; North Rice, 2013, 43(6): 26–28

Note: The 17 experimental datasets collected in Fujian province do not include data from experimental sites in Pinghe county and Xianyou county.

### Construction of the TNFM

The mathematical expression of the unary quadratic polynomial fertilizer response model used in this study is: Y = *b*_0_ + *b*_1_X + *b*_2_X^2^, where Y is the fitting crop yield; X is the application rate of N, P_2_O_5_, or K_2_O fertilizer; and *b*_0_, *b*_1_ and *b*_2_ are the fertilizer response coefficients.

To address the problems of specification bias and multicollinearity in the quadratic polynomial fertilizer response model^8^, Zhang *et al*.^16^ established a unary non-structural fertilizer response model:

$${\rm{Y}}=A({s}_{0}+{\rm{X}}){e}^{-c{\rm{X}}},$$

where Y is the crop yield; X is the application rate of N, P_2_O_5_, or K_2_O fertilizer; *s*_0_ is the equivalent of soil supplying nutrient; *c* is the yield coefficient of fertilization; and *A* is the conversion coefficient of soil fertility to rice yield at X = 0, which comprehensively reflects the soil productivity. Therefore, a TNFM can be described according to the principle of irreplaceable function of plant nutrient elements as:

$${\rm{Y}}={A}_{N}({N}_{0}+{\rm{N}}){e}^{-{c}_{1}{\rm{N}}}\times {A}_{P}({P}_{0}+{\rm{P}}){e}^{-{c}_{2}{\rm{P}}}\times {A}_{{\rm{K}}}({K}_{0}+{\rm{K}}){e}^{-{c}_{3}{\rm{K}}},$$

where *N*_0_, *P*_0_, and *K*_0_ are the soil nutrient supply equivalents of N, P_2_O_5_ and K_2_O, respectively, and *c*_1_, *c*_2_ and *c*_3_ are the yield increase effect coefficients for nitrogen, phosphorus and potash fertilizer, respectively. The meanings of *A*_N_, *A*_P_ and *A*_K_ are similar to that of *A* in model (2), and the meanings of the other algebraic symbols are the same as that in model (2). The formula can be further converted into the TNFM:

$${\rm{Y}}=A({N}_{0}+{\rm{N}})({P}_{0}+{\rm{P}})({K}_{0}+{\rm{K}}){e}^{-{c}_{1}{\rm{N}}-{c}_{2}{\rm{P}}-{c}_{3}{\rm{K}}},$$

where *A* = *A*_N_ × *A*_P_ × *A*_K_ is the conversion coefficient for soil fertility to rice yield when application rates of nitrogen, phosphorus and potassium fertilizer equal zero.

### Parameter estimation and statistical testing of the TNFM

Model (3) is a nonlinear model that cannot be directly linearized, so the model parameters are estimated by the use of a nonlinear least squares method^27^. If the nonlinear fertilizer response model is Y = *f* (*X*, *a*), the nonlinear least squares problem can be solved to obtain an estimated value of the parameter *a*:

6 $$min\,Q(a)=\sum _{i=1}^{n}{({Y}_{i}-f({X}_{i},a))}^{2}$$

The solution $\hat{a}$ is an estimated value of the parameter *a*. The regression significance test of model (3) is similar to that for the TPFM, but the degrees of freedom for the regression are 6. In this paper, we used the performance function “nlinfit” in the MATLAB software (https://cn.mathworks.com/programs/trials/trial_request.html) to conduct the parameter estimation and statistical test of the TNFM, and the performance function “regress” was used for the regression analysis of the TPFM. Graphs were drawn with the MATLAB programming language. The mathematical principles of concrete calculation and the use of relevant performance functions can be found in the relevant monographs^27,28^.

### The typicality discrimination method for a ternary fertilizer response model

The typicality of a fertilizer response model involves evaluating the reliability of fertilization recommendations by the marginal product derivative method. Because of the complexity of agricultural production conditions, the equation effect curve or surface has a great diversity of shapes^13,14^ in the fertilizer response models created from the results of field experiments. Zhang *et al*.^15^ reported that one typical model and three types of non-typical models exist for a TPFM according to passing a significance test.

A typical TPFM can satisfy the following conditions at the same time: (1) all algebraic signs of monomial coefficients are positive numbers, and all the algebraic signs of the quadratic coefficients are negative numbers, (2) there is a global maximum output point in the fertilizer response model, and (3) both the maximum fertilization rate and economic fertilization rate estimated by the marginal product derivative method fall into the range of fertilization rates in the experimental design. Such a fertilizer response model is designated as a typical fertilizer response model because it conforms to the general fertilizer response rule of plant nutrition. The marginal product derivative method can be used for fertilization recommendations. Otherwise, if any one of the three conditions could not satisfied, the model would be designated as a non-typical fertilizer response model, which belongs to the types of the unreasonable coefficient signs model or the no maximum yield point model or the extrapolation fertilization recommendations rate model, respectively. It indicates that the fertilization recommendations rate is unreliable with the marginal product derivative method.

How can the existence of a global maximum yield point in the ternary quadratic polynomial fertilizer response model be assessed? According to an unconstrained optimization method^29^, if the first-order gradient vector quantity g (X*) of a fertilizer response model at a point X* (X* = (N, P, K) vector) is equal to the zero vector, and the determinants of principal minors in its Hesse matrix G(x) are: G_1_ = 2b_4_; G_2_ = 4b_4_b_5_ − b_7_^2^; G_3_ = 2(4b_4_b_5_b_6_ + b_7_b_8_b_9_ − b_4_b_9_^2^ − b_5_b_8_^2^ − b_6_b_7_^2^), then (1) if g(X*) = 0, and G_1_ < 0, G_2_ > 0, G_3_ < 0, the Hesse matrix g(X) is negative-definite and the model has a global maximum output point. (2) If g(X*) = 0, and G1 > , G2 > 0, G3 > 0, the Hesse matrix g(X) is positive-definite and the model has a global minimum output point. (3) If g(X*) = 0, G_1_, G_2_ and G_3_ do not meet the conditions for the positive-definite and negative-definite of the Hesse matrix G (x), and are not equal to zero, then the Hesse matrix is indefinite and no maximum output point exists in the model.

Given that a requisite test of significance is passed, the TNFM may also have different types of models: (1) if all of the model parameters such as *A*, *N*_0_, *P*_0_, *K*_0_, *c*_1_, *c*_2_ and *c*_3_ are greater than zero, the maximum fertilization rates and economic fertilizer rates of N, P and K fertilizers fall into the range of the fertilization rate in an experimental design, and the model satisfies the general fertilizer response law of plant nutrition, then the model could be designated as a typical fertilizer response model. But (2) if one or more of the model coefficients including *A*, *N*_0_, *P*_0_, *K*_0_, *c*_1_, *c*_2_ and *c*_3_ are negative, the model does not satisfy the general law of plant nutrition and the model could be designated as a non-typical model of a type that contains unreasonable coefficient signs. However, (3) if all of the model parameters *A*, *N*_0_, *P*_0_, *K*_0_, *c*_1_, *c*_2_ and *c*_3_ are greater than zero, but either one or both of the maximum fertilization rate or economic fertilizer rate recommended by the marginal product derivative method falls outside the range of the fertilization rate in an experimental design, the model could be designated as a non-typical model of the type for which a fertilization rate could be recommended by extrapolation. Because of the mathematical structural characteristics of the unstructured model, if the coefficients mentioned above are greater than zero, a global model maximum yield point would surely exist. Thus, no non-typical model that does not have a maximum yield point can be characterized as a ternary non-structural fertilizer response model.
